# Giving Bad Science the Stamp of Approval: Policy and Legal Consequences of a Vaccine Scare in Italy

**DOI:** 10.3389/phrs.2024.1606756

**Published:** 2024-02-14

**Authors:** Marco Rizzi, Katie Attwell

**Affiliations:** ^1^ UWA Law School, University of Western Australia, Perth, WA, Australia; ^2^ School of Social Sciences, University of Western Australia, Perth, WA, Australia

**Keywords:** vaccine hesitancy, public health, vaccine mandates, health policy, health law

## Introduction

Twelve years ago, a decision by an Italian court catalyzed a series of events leading to new mandatory childhood vaccination laws in 2017. As vaccine misinformation and mandates continue to animate public debates globally, Italy’s experience remains a pertinent cautionary tale on the risks of vaccine misinformation infiltrating institutions [1–3].

Misinformation works its way into public discourse in many ways, the most insidious of which takes a fraudulent theory and cloaks it in scientific and institutional authority. Debunking that theory then threatens the credibility of the entire system. This is what happened when Italian courts linked MMR vaccines and autism [4]. The incident contributed to vaccine hesitancy and refusal, eventually leading authorities to adopt mandates amid resultant disease outbreaks.

## Bad Science in Court

In 1998, Wakefield indirectly linked MMR vaccination to autism in the *Lancet*, forging a powerful connection between the two in public discourse. The journal retracted the fraudulent article in 2010, yet it informed two judgments issued by Italian courts in 2012 in the town of Rimini, and in 2014 in Milan.

The Rimini case saw the family of an autistic child lodge a claim through a government compensation scheme, alleging that the child’s autism had been caused by MMR vaccination. The State Attorney’s Office, representing Italy’s Ministry of Health, relied exclusively and unsuccessfully on a procedural defence. When the case moved to a hearing, the government lawyers did not attend. The court-appointed expert supported the plaintiff’s claim, referring to Wakefield’s paper 2 years after its retraction [1, 5].

In Milan, a similar situation arose for the hexavalent vaccine (a single combination vaccine against diphtheria, tetanus, acellular pertussis, *Haemophilus* influenzae type B, poliovirus, and hepatitis B). There has never been suggestion of an autism link with this vaccine. Here, the State Attorney failed to back their defence with expert testimony, and the court-appointed expert found the vaccine to be the most probable cause of the child’s autism [1].

Both cases were overturned on appeal, but their social impact was enormous, and Italian authorities were unprepared for the aftermath [2–4].

## From Bad Judgments to a Measles Outbreak

Attaining high levels of immunity in populations requires governments to deliver mass vaccination programs that perennially re-embed vaccination as the social norm. Among other key ingredients, vaccination drives need messaging to inform, inspire, and overcome concerns about safety or efficacy [6].

In the early 2000s, Italy’s national and regional governments were confident in their ability to implement a voluntary vaccination program. In 2007, the Veneto region removed existing childhood vaccine mandates (for polio, diphtheria, tetanus, and hepatitis B), adopting instead a sophisticated communication campaign. There was confidence in the population’s ability to make the “right” decisions. After its initial success in retaining high coverage rates, the Veneto pilot scheme was intended for national replication. The rest of Italy was still ostensibly mandating these vaccines but with limited enforcement and penalties—school requirements had been overturned in 1999 and the remaining penalty of fines was rarely applied [4].

The 2012 Rimini decision caught authorities off guard. Google search data show spikes of searches for the MMR-autism link in the aftermath and the following years [3]. Italy’s vaccine hesitancy problem needed a national approach, but the Ministry of Health had limited capacity, and its personnel did not know how to engage with misinformation in the emergent online world [4].

Moreover, austerity had stretched the Italian public service following the 2008 Global Financial Crisis. Elected officials had low levels of interest and scarce resources to invest in the vaccination problem. As MMR vaccination rates fell, Ministry staff appealed unsuccessfully for funds to address misinformation with communications campaigns. As the crisis unfolded, the crucial voice of government was ineffective [4].

MMR vaccination rates dropped from 90% in 2013 to 85% in 2015, in contrast to comparable countries like France and Germany where rates remained above 90% throughout the same period. In 2017 Italy experienced over 5,000 cases of measles [7].

## Mandates as an Enduring Policy Response

**FIGURE 1:**
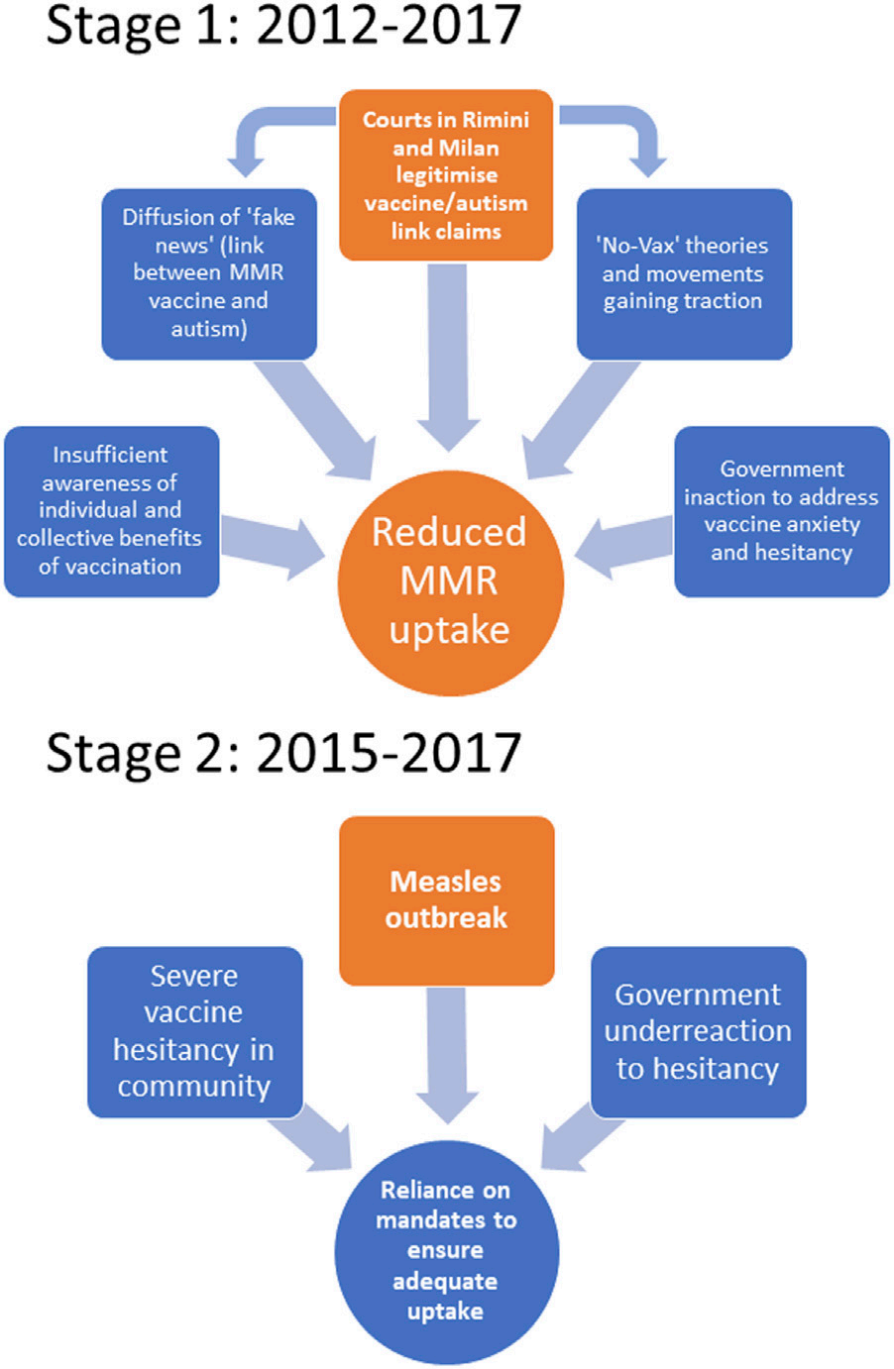
Lead up to 2017 Italian mandates in 2 stages (Australia, 2020-2023).

Regional and national governments reached for mandates in response. Veneto suffered the biggest decline in MMR vaccination rates in the country, and somehow it seemed as though having *no* mandates was contributing to MMR vaccine refusal there. National officials drew neat conclusions: vaccine mandates work, removing them is dangerous, and populations cannot be trusted to make “good” decisions. They also saw mandates as an effective way for government to demonstrate support for vaccination to the public [4]. Yet mandates can be controversial or backfire, and there are benefits in pursuing more voluntaristic policies first [8]. Italy eventually delivered an online campaign about the importance of vaccination, but still resorted to mandating ten vaccines (including MMR) in 2017 (Figure 1). These mandates remained in place despite fierce political opposition to the policy, including within government, as the country dealt with a further measles outbreak in 2019 [4].

## The Italian Lesson

The Rimini and Milan court cases illustrate several problems within Italy’s legal and administrative systems that may pose similar risks elsewhere. These include ineffective communication between responsible ministers and their lawyers, a lack of resources and staffing, and insufficient awareness amongst government stakeholders of the sensitivity of public health issues [1].

A key lesson is that it is crucial to equip the process and the relevant personnel involved in judicial truth-finding with adequate mechanisms for verifying scientific evidence. In the United States, courts play a gatekeeping role by assessing the soundness of expert witnesses’ reasoning and method. The US system is imperfect and subject to heavy criticism, but it forces courts to appraise the quality and credibility of scientific witnesses and their evidence before engaging with their substance [9]. By contrast, European courts “co-produce” judicial truth through collaboration between the expert and the judge who appoints them. The focus is on their relationship of trust and confidence, rather than on clear expertise-based appointment criteria. Indeed, the experts appointed in the Rimini and Milan cases were forensic pathologists with no specialist knowledge of vaccines or immunology [1]. In this context, the resultant “judicial truth” can depart from a rigorous application of the scientific method, producing spurious or misguided results. The risk of the system being seriously affected by misinformation is real, and decisions like those in Rimini and Milan invite greater scrutiny of the specialist expertise of individuals tasked with assisting judges.

A second lesson from Italy is that legal and political systems must be cognizant of the broader impact of controversial cases. Wakefield’s theory rose and died in the scientific world but found new life in Italian society when the Rimini and Milan decisions bestowed it with legitimacy. It became a dangerous “zombie idea” that contributed to the stirring of social moods, the corresponding rise of vaccine hesitancy and, ultimately, the shaping of reactive government strategies [1].

A third lesson is that legal processes alone cannot ensure that “the truth will out.” Adequate resources need to be invested in shaping public discourse. The Rimini and Milan decisions did not create damaging legal precedents, as they were overturned on appeal. However, they generated real public health and governance problems long after being overturned. They facilitated the dissemination of anti-vaccination theories; provided false hope of economic relief and vindication to families of autistic children; and—exacerbated by public communication failures—further nudged Italy towards vaccine mandates [4, 5]. There is of course no neat causal link between the Rimini and Milan decisions and the adoption of mandates in 2017. But the decisions were important threads in the complex tapestry that ultimately produced that result [1].

The endpoint of this story is that Italy is now “one of the countries that most embodies the ‘mandatory’ approach to childhood vaccinations” [10]. Mandates have their strengths: they can help governments attain high coverage rates and protect the public against disease outbreaks. In a pandemic, like COVID-19, they might even be necessary. But they can also symbolize a government’s earlier failures to build and maintain community support for vaccination.

Italy’s “vaccines cause autism” case is certainly extreme. Yet, it stands as a reminder that successful governance of vaccine programs requires political and technical actors to be alive to all threats to vaccine confidence. At a minimum, they must avoid bestowing unwarranted stamps of legitimacy upon misinformation.
